# Mitogen-Activated Protein Kinase 4-Regulated Metabolic Networks

**DOI:** 10.3390/ijms23020880

**Published:** 2022-01-14

**Authors:** Chuwei Lin, Aneirin Alan Lott, Wei Zhu, Craig P. Dufresne, Sixue Chen

**Affiliations:** 1 Department of Biology, University of Florida, Gainesville, FL 32611, USA; chuwei.lin@ufl.edu (C.L.); a.lott@ufl.edu (A.A.L.); zhuwei2007100@163.com (W.Z.); 2 University of Florida Genetics Institute (UFGI), University of Florida, Gainesville, FL 32610, USA; 3 Plant Molecular and Cellular Biology Program, University of Florida, Gainesville, FL 32610, USA; 4 Institute of Cancer and Basic Medicine, Chinese Academy of Sciences, Hangzhou 310002, China; 5 Thermo Fisher Scientific, 1400 Northpoint Parkway, West Palm Beach, FL 33407, USA; craig.dufresne@thermofisher.com; 6 Proteomics and Mass Spectrometry, Interdisciplinary Center for Biotechnology Research (ICBR), University of Florida, Gainesville, FL 32610, USA

**Keywords:** *Arabidopsis thaliana*, mitogen-activated protein kinase 4, metabolomics and proteomics, immunity, cytokinesis, polyamine metabolic networks

## Abstract

Mitogen-activated protein kinase 4 (MPK4) was first identified as a negative regulator of systemic acquired resistance. It is also an important kinase involved in many other biological processes in plants, including cytokinesis, reproduction, and photosynthesis. *Arabidopsis thaliana* *mpk4* mutant is dwarf and sterile. Previous omics studies including genomics, transcriptomics, and proteomics have revealed new functions of MPK4 in different biological processes. However, due to challenges in metabolomics, no study has touched upon the metabolomic profiles of the *mpk4* mutant. What metabolites and metabolic pathways are potentially regulated by MPK4 are not known. Metabolites are crucial components of plants, and they play important roles in plant growth and development, signaling, and defense. Here we used targeted and untargeted metabolomics to profile metabolites in the wild type and the *mpk4* mutant. We found that in addition to the jasmonic acid and salicylic acid pathways, MPK4 is involved in polyamine synthesis and photosynthesis. In addition, we also conducted label-free proteomics of the two genotypes. The integration of metabolomics and proteomics data allows for an insight into the metabolomic networks that are potentially regulated by MPK4.

## 1. Introduction

Mitogen-activated protein kinases (MAPKs) are conserved protein kinases in eukaryotes. In plants, MAPK signaling cascades are involved in multiple cellular processes in response to environmental and developmental stimuli. After activation, MAPKs phosphorylate downstream proteins to regulate plant growth, development, and defense responses. There are 20 MAPKs in the genome of reference plant *Arabidopsis thaliana* [1]. Since the *mpk4* homozygous mutant displays a severe dwarf phenotype with curly and small leaves and short and swollen root tips, MPK4 appears to be important for plant cell division, morphogenesis, and development [2]. In fact, MPK4 has been shown to be involved in several biological processes, including cytokinesis [3,4,5,6], reproduction [7], growth [8], and photosynthesis [9]. Two distinct processes in cytokinesis, i.e., microtubule (MT) transition and septum formation, were found defective in the *mpk4* mutant [4,5,6]. Multinucleate cells are more frequently observed in *mpk4* cotyledon epidermal cells than wild-type (WT) plants, indicating incomplete phragmoplast expansion [5]. MTs in *mpk4* root hairs show a severe aberrant phenotype, disoriented arrays, and thicker bundling, compared with parallel organization in WT [4].

Co-immunoprecipitation between MPK4 and the microtubule-associated protein MAP65-1 also indicates that MPK4 is related with MTs. Non-phosphorylated MAP65-1 form is increased in the *mpk4* mutant, which also provides evidence that MAP65-1 is a potential substrate of MPK4 [4]. MPK4 can also phosphorylate a newly synthesized septum-enriched protein, PATELLIN2 (PATL2), a regulator of an auxin efflux carrier PIN [6]. Abnormal cytokinesis during male meiosis in plants also leads to the sterilization of *mpk4* [2,7]. Pollen grains of *mpk4* are largely nonviable, with a larger size but fewer grains as a result of failed meiosis [7]. Cytokinins are a class of phytohormones involved in plant cell cytokinesis, such as kinetin and zeatin. Cytokinins influence cytokinesis by guiding MT dynamics and organization [10]. They also increase seed yield by promoting ovule size and reproductive meristem activity [11]. Moreover, it stimulates the lytic degradation of PIN1 during organogenesis [12]. In addition to cytokinins, there are other metabolites mediating plant growth and reproduction, such as polyamines [13,14].

MPK4 was first identified as a negative regulator of systemic acquired resistance (SAR). Salicylic acid (SA) is a crucial phytohormone involved in plant SAR signaling pathways. It accumulates during avirulent pathogen invasion and binds with NON EXPRESSOR OF PR1 (NPR1, *At1g64280*), a receptor of SA, inducing pathogen defense [15]. In the *mpk4* mutant, the levels of SA and its derivative salicyl acyl glucuronide (SAG) increased compared with WT plants, leading to a resistance to *Pseudomonas syringae* pv. *tomato* (*Pst*) DC 3000 [2]. Meanwhile, the constitutively active MPK4 (CA-MPK4) mutant showed a lower SA level, resulting in a susceptibility to the same pathogen [16]. Consistent with these results, under ozone exposure the SA level increased in *MPK4*-silenced plants [17]. However, in *mpk4/eds1* and *mpk4/pad4* double mutants, the accumulation of SA is relieved, indicating that MPK4 may suppress SA accumulation by inhibiting EDS1 and PAD4 [18]. Jasmonic acid (JA) and its methyl ester jasmonate (MeJA) are important plant hormones that are also involved in plant immunity. They are induced by wounding caused by herbivores and often counteract SA [19,20,21]. For example, in the *mpk4* mutant, JA induced the expression of *THI2.1,* and *PDF1.2* genes was blocked [2]. Contrary to expectation, in *MPK4*-overexpression plants, JA and MeJA levels were decreased [22]. In addition, MPK4 showed kinase activity after wounding, indicating that MPK4 may also be involved in the JA signaling pathway [17]. However, the levels of JA and JA-related metabolites in the *mpk4* mutant have not been analyzed.

There are about 1 million metabolites in plant kingdoms by estimation [23,24]. Metabolites, as end products of enzyme activities, have diverse biochemical and physiological properties and vary in concentrations from femtomolar to millimolar. They form intricate cellular molecular networks that determine plant chemotype and phenotype [25,26,27]. Metabolites can be divided into primary and specialized metabolites. Primary metabolites are related to the intrinsic function of plants, including growth, development, and reproduction. They are essential to the survival of plants. Compared to primary metabolites, specialized metabolites do not directly affect the essential functions of plants, but enhance the fitness under biotic and abiotic stresses. Depending on the cell and tissue types, metabolome varies because of different internal and external factors, such as epigenetic marks and environmental stresses. Compared to analyses of transcriptome and proteome, metabolomic characterization has been challenging due to the vast diverse chemical structures. There are only 4806 metabolite records in PlantCyc and 2854 in KEGG. Metabolomics, the study of the whole set of metabolites, provides an approach to identify and quantify those important phytochemicals in plants. There are two complementary metabolomics approaches, targeted metabolomics and untargeted metabolomics. Targeted metabolomics is based on previous experiments and literature. It makes it possible to precisely identify and quantify known metabolites from large sample sets. Often limited by the availability of authentic compounds, targeted metabolomics enables the analysis of several hundreds of metabolites [28]. On the other hand, untargeted metabolomics can reveal thousands of metabolite features, but only a small number may be structurally annotated. Untargeted metabolomics is challenged by the accurate chemical annotation of all observable features and limited depth-of-coverage. Trapped ion mobility spectrometry (TIMS) coupled to quadrupole time-of-flight tandem mass spectrometry (timsTOF-MS/MS) provides an additional dimension of separation compared to traditional MS, increasing the depth-of-coverage. Metabolite identification confidence can be enhanced by increased peak capacity and collision cross section (CCS) data matching [29]. This study aims to profile metabolites and their differences between *A. thaliana* WT plants and the *mpk4* mutant. Adding to previous transcriptomics and proteomics datasets, multi-omics data provide new insights into understanding how MPK4 affects plant metabolic networks and dynamic changes.

## 2. Results

### 2.1. Mpk4 Mutant Phenotype and Transcription Analysis

**Figure 1:**
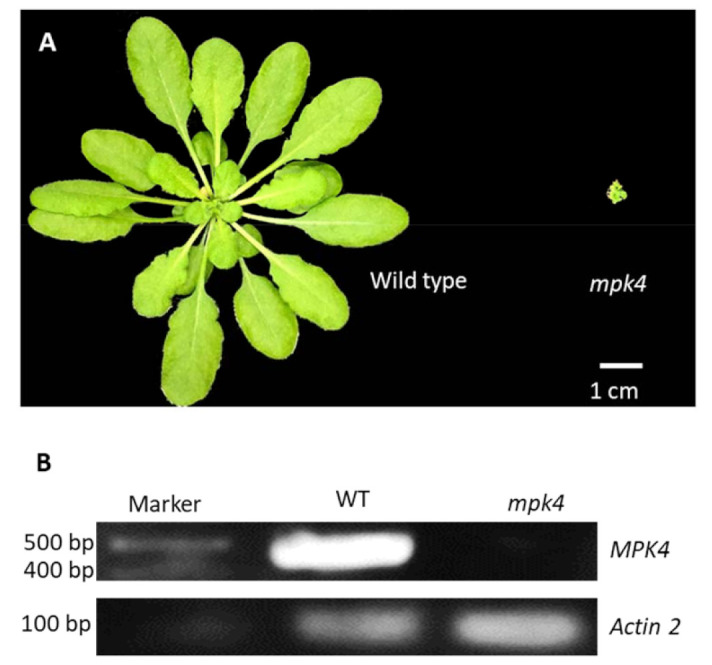
Morphological phenotype and transcript analysis of *mpk4*. (**A**) 5-week-old *Arabidopsis thaliana* wild-type (WT) (left) and *mpk4* mutant (right). (**B**) Semi-qPCR result showing abundant *MPK4* transcript in WT, but none in the *mpk4* mutant.

A five-week-old *mpk4* mutant plant was a severe dwarf with curling leaves. Mature leaves of WT Col-0 plants were about 2.5 cm long, while the entire rosette diameter of the *mpk4* mutant was about 0.5 cm (Figure 1A). A semi-qPCR result showed that *mpk4* homozygous did not accumulate any detectable *MPK4* transcript, while the WT had a high level of *MPK4* expression (Figure 1B). These results are consistent with previous studies [2,18,30].

### 2.2. Metabolomics of WT and the Mpk4 Mutant Leaves

**Figure 2:**
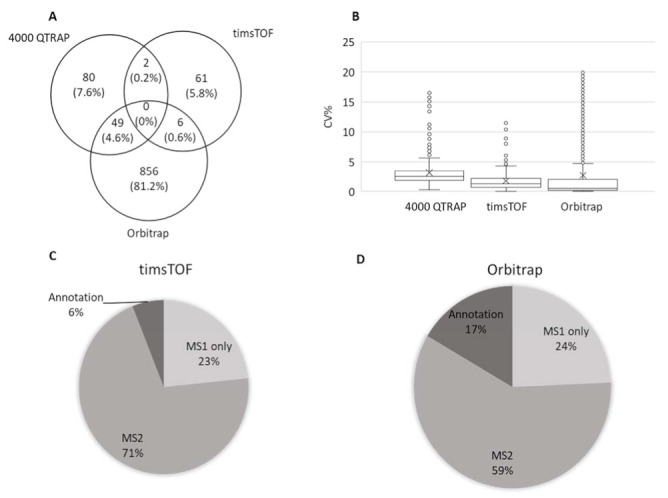
Comparison of metabolomics data from three different mass spectrometry platforms. (**A**) Venn diagram showing overlap of identified metabolites from targeted (4000 QTRAP) and untargeted metabolomics (timsTOF and Orbitrap). (**B**) coefficient of variance (CV%) of the quantitative data from the three platforms. (**C**) timsTOF data composition. (**D**) Orbitrap data composition. MS1 indicates features only have MS1 spectra and do not have annotation; MS2 indicates features have both MS1 and MS2 spectra but no annotation; annotation indicates those metabolites with both MS1 and MS2 spectra, and annotated with names and chemical formulars.

Metabolic profiles of WT and *mpk4* were analyzed using the targeted metabolomics method based on HPLC-MRM-MS (4000 QTRAP platform), and untargeted metabolomics methods based on timsTOF-MS and Orbitrap MS platforms. In total, 1776 metabolites were identified with chemical names and structures (Table S1), two were shared by targeted and timsTOF, 49 were shared by targeted and Orbitrap, and six were shared by timsTOF and Orbitrap (Figure 2A).

According to metabolite identification confidence levels for global untargeted molecular omics [31,32], there were 131 annotated metabolites profiled in targeted metabolomics at level 1, 69 in untargeted metabolomics with timsTOF at level 2, and 911 with the Orbitrap at level 2. Comparing the three platforms, means of coefficiency of variance were all less than 5%, while data from Orbitrap showed smallest variance among the four replicates, followed by timsTOF and then 4000 QTRAP (Figure 2B). When comparing the two untargeted platforms, timsTOF and Orbitrap, Orbitrap detected 16,139 total features, while timsTOF detected 10,577 total features. Among the features detected by timsTOF, 23% had only MS1 spectrum, 77% had MS2 but no annotation, and 6% were annotated with names and structures (Figure 2C). While in the MS data from the Orbitrap, 24% had only MS1, 59% had MS2 but no annotation, and 17% had annotation with names and structures (Figure 2D). Databases, including mzCloud^TM^, ChemSpider, and Metabolika, were used for data acquired from the Orbitrap contribute to the increase of chemical annotations. Considering that different metabolomics libraries were used for data acquired from the different platforms, it is hard to have a fair comparison between the two untargeted metabolomic datasets. Here we took advantage of the hyphenated metabolomics platforms and combined the three sets of data. Redundant metabolites were compared between three platforms based on their CV. Those with the smallest CV were used for further statistical analysis.

**Figure 3:**
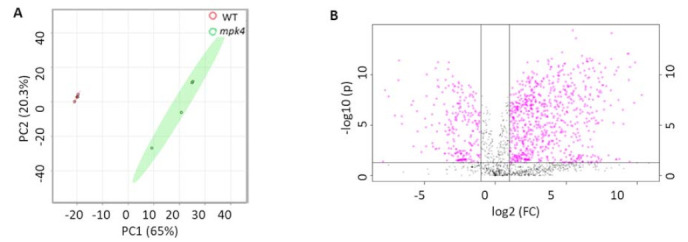

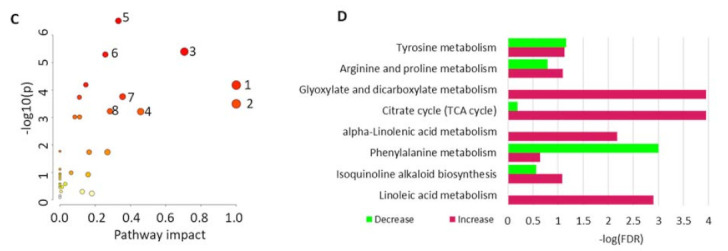
Metabolomics data analysis of WT and *mpk4* mutant. (**A**) PCA analysis showing distinct separation of WT and the *mpk4*. (**B**) Volcano plot. FC (fold change) = metabolite abundance in *mpk4*/WT. (**C**) KEGG metabolic pathway impact analysis. 1: linoleic acid metabolism; 2: isoquinoline alkaloid biosynthesis; 3: phenylalanine metabolism; 4: alpha-linolenic acid metabolism; 5: citrate cycle; 6: glyoxylate and dicarboxylate metabolism; 7: arginine and proline metabolism; 8: tyrosine metabolism. The size of the dot indicates pathway impact factor; color of dots from light yellow to red indicates—log10 (p) from small to large. (**D**) KEGG enrichment analysis of eight significant pathways.

**Figure 4:**
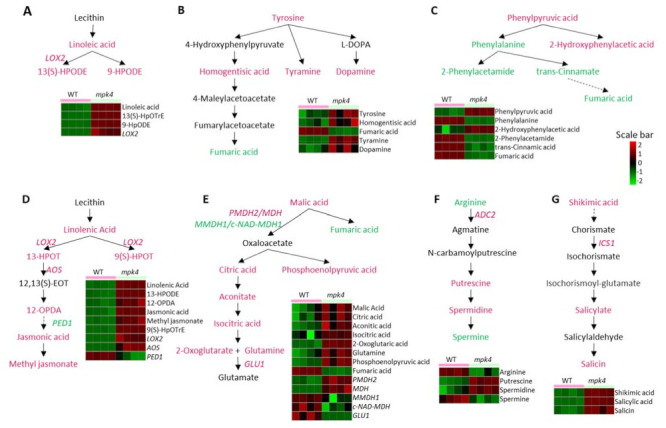
Pathways with differential metabolites and proteins between WT and the *mpk4* mutant. (**A**) Linoleic acid metabolism. (**B**) Tyrosine metabolism. (**C**) Phenylalanine metabolism. (**D**) Alpha-Linolenic acid metabolism. (**E**) Citrate cycle. (**F**) Arginine and proline metabolism. (**G**) Salicylic acid synthesis. red: increased in *mpk4*; green: decreased in *mpk4*; solid arrow: direct reaction; dotted arrow: multiple reaction steps.

Principal component analysis (PCA) of the metabolomics data from the four replicates showed that the data from each phenotype were grouped together, and the WT and *mpk4* were classified into distinct clusters (Figure 3A). The significantly different metabolites were selected based on the criteria of a fold change > 2 and *p-*value < 0.05. A total of 898 differential metabolites were identified between the WT and *mpk4* (Table S2). Among them, 123 metabolites decreased in *mpk4*, while 517 increased significantly (Figure 3B). However, only 183 metabolites were found in the KEGG database that were assigned for KEGG Metabolomic Pathway Analysis (MetPA) [33]. Metabolites that were not in KEGG databases were not used for pathway analysis. For example, N-hydroxypentahomomethioninate, s-methyl methanesulfinothioate, 1-Ethylsulfinylsulfanylethane, tetrahydrogeranylgeranyl-PP, and 2,2’-bipyridine can be found in AraCyc, but do not have corresponding KEGG entries or any literature information about their functions in *A. thaliana*. Among the 34 KEGG pathways with metabolite hits, eight pathways were significantly impacted (*p*-value < 0.05 and impact factor > 0.2). They include linoleic acid metabolism, isoquinoline alkaloid biosynthesis, phenylalanine metabolism, alpha-linolenic acid metabolism, citrate cycle, glyoxylate and dicarboxylate metabolism, arginine and proline metabolism, and tyrosine metabolism (Figure 3C). Pathway enrichment analysis of the eight pathways showed that the phenylalanine metabolism pathway mainly decreased, while other pathways mainly increased (Figure 3D). In linoleic acid metabolism, linoleic acid, 13(S)-hydroperoxy-(9Z,11E)-octadecadienoic acid (13(S)-HPODE), and 9-hydroperoxy-(10E,12E)-octadecadienoic acid (9-HPODE) were increased (Figure 4A). Tyrosine, homogentisic acid, tyramine and dopamine from tyrosine metabolism increased (Figure 4B). Phenylalanine, 2-phenytacetmide, trans-cinnamate, and fumaric acid from phenylalanine metabolism decreased (Figure 4C). In alpha-linolenic acid metabolism pathway, linolenic acid, (9Z,11E,13S,15Z)-13-hydroperoxyoctadeca-9,11,15-trienoic acid (13-HPOT), (10E,12Z,15Z)-(9S)-9-hydroperoxyoctadeca-10,12,15-trienoic acid (9(S)-HPOT), and 12-oxophytodienoic acid (12-OPDA) were increased (Figure 4D). Malate, citrate, phosphoenolpyruvate, cis-aconitate, isocitric acid, and 2-oxoglutarate, involved in glyoxylate and dicarboxylate metabolism as well as the citrate cycle, were all increased (Figure 4E). In arginine metabolism, arginine and spermine decreased, while putrescine and spermidine increased (Figure 4F). JA, downstream of 12-OPDA, was also increased. In addition to these pathways, shikimic acid and salicin, involved in salicylate biosynthesis, as well as salicylate (Figure 4G), were increased in *mpk4*, which is consistent with the previous results [18].

### 2.3. Proteomics of WT and the Mpk4 Mutant Leaves

**Figure 5:**
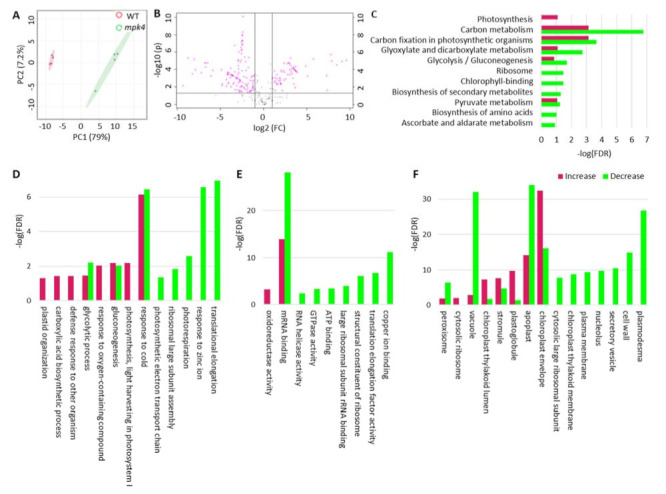
Proteomics data analysis of WT and the *mpk4* mutant. (**A**) PCA analysis. (**B**) Volcano plot. FC (fold change) = protein level in *mpk4*/WT. (**C**) KEGG pathway enrichment analysis. (**D**) GO biological process. (**E**) GO molecular function. (**F**) GO cellular component.

Using label-free quantitative proteomics, a total of 8048 MS/MS spectra corresponding to 865 proteins were identified through the LC-MS/MS and TAIR 10 database searching with Proteome Discoverer 2.5. Identified proteins were filtered with unique peptides numbered larger or equal to 2 and FDR < 0.01. Based on PCA, four replicates of each phenotype were grouped together and classified into distinct clusters (Figure 5A). A twofold change cut-off and *p*-value of < 0.05 were used to determine significant changes in the levels of proteins between the WT and *mpk4*. A total of 184 proteins were found to be differentially expressed (Table S3), with 64 increased and 120 decreased in the *mpk4* compared with WT (Figure 5B) (Table S4). The differential proteins were used for KEGG pathway analysis to understand protein functions in metabolic pathways. Among the 72 pathways with protein hits, nine were significantly impacted with *p*-values < 0.05, including ascorbate and aldarate metabolism, biosynthesis of amino acids, pyruvate metabolism, photosynthesis—antenna proteins, glycolysis/gluconeogenesis, ribosome, glyoxylate and dicarboxylate metabolism, carbon fixation in photosynthetic organisms, and photosynthesis pathways (Figure 5C). Gene ontology (GO) functional classifications including biological process, cellular component, and molecular function were also generated based on differential proteins (Figure 5D). According to GO biological process analysis, the reactive oxygen species metabolic process decreased in *mpk4* mutant, which was consistent with previous studies in which the ROS scavenging mechanism was disrupted in *mpk4* [34,35]. According to GO molecular function analysis, oxidoreductase activity increased in *mpk4*, and activities such as copper ion binding and translation elongation factor activity decreased. According to GO cellular components, proteins located on plastoglobuli, chloroplast thylakoid lumen, and chloroplast envelop significantly increased in *mpk4*, which was consistent with the observation that *mpk4* had an increasing number of plastoglobuli [9].

### 2.4. Integration of Metabolomics and Proteomics

**Figure 6:**
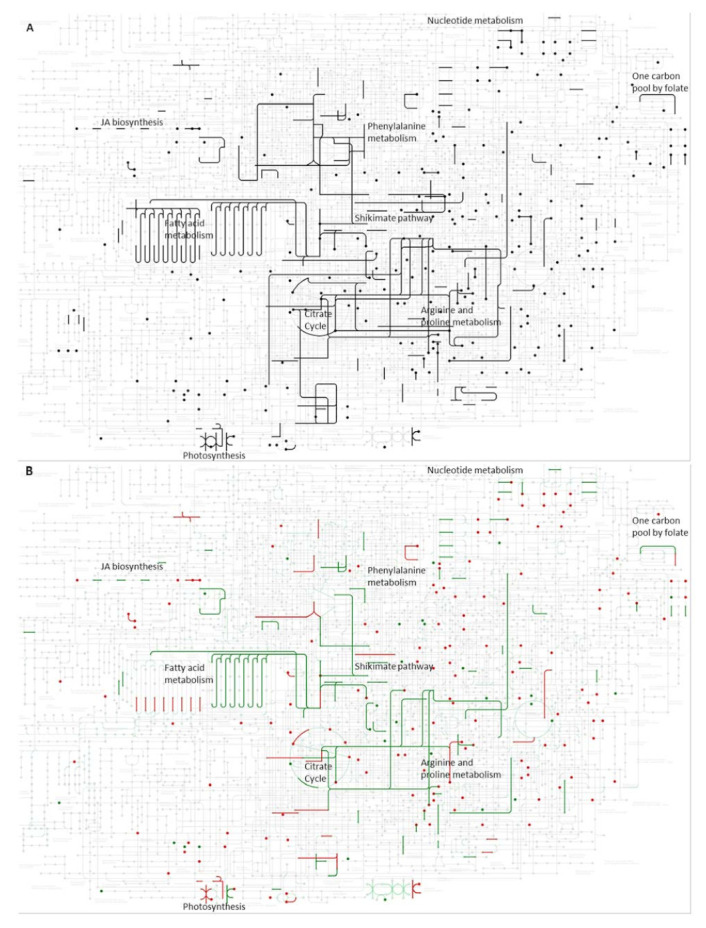
Integrated metabolite and protein network shown in KEGG pathways. (**A**) All metabolites and proteins found in samples are shown in KEGG pathway map. Dots indicate metabolites and lines indicate proteins. (**B**) Metabolites and proteins with significant changes (>twofold and *p* < 0.05) are shown in KEGG pathway map. Green indicates decreased in the *mpk4* and red indicates increased in the *mpk4*. Please refer to Figure S1 for a high-resolution version of the pathways.

To associate the results of metabolomics and proteomics, the identified metabolites and proteins were integrated into a KEGG pathway map (Figure 6A). Proteins and metabolites with significant changes were colored (Figure 6B).

Proteins and metabolites found in both WT and the *mpk4* mutant mostly cover carbohydrate metabolism, lipid metabolism, and amino acid metabolism pathways. Among these pathways, changed proteins and metabolites share seven pathways, including linoleic acid metabolism, tyrosine metabolism, phenylalanine metabolism, alpha-linolenic acid metabolism, citrate cycle, arginine and proline metabolism, and salicylic acid synthesis, which were selected and analyzed (Figure 4). Except the arginine biosynthesis pathway, most proteins and metabolites from other pathways increased in *mpk4*, indicating protein level regulation. Pearson correlation between metabolite contents and protein abundances of corresponding KEGG pathways was used to understand biochemical pathway activities and regulations (Table S5).

## 3. Discussion

### 3.1. Comparison between Different Metabolomic Platforms

In this study, we integrated both metabolomic and proteomic changes attributed to the mutation of MPK4, an important kinase known to function in plant defense, growth, and development [2,3,4,5,6,7,8,9]. In terms of the two untargeted metabolomic platforms (timsTOF and Orbitrap), timsTOF provided the same percentage of MS2 spectra among the total features as Orbitrap. However, it only had 6% features annotated with chemical names and structures. This may be attributed to the fact that the raw files from timsTOF could not be processed through other databases and libraries, such as mzCloud^TM^, and that the Bruker MetaboBASE library size is limited. Although the ion mobility data can be useful, libraries with CCS values are very limited. In addition, these raw files were incompatible with other search engines, such as MZmine and the Thermo Compound Discoverer. This is also the reason why timsTOF data had the least overlap with the other two sets of data. A generic database/library format that can be used by different vendor’s software may greatly enhance the metabolite annotation. With a gradual improvement of the metabolite databases with more and more CCS data, timsTOF-based plant metabolomics datasets should get more chemical annotations in the future.

### 3.2. MPK4 Is Involved in Arginine Metabolism, JA and SA Changes

Based on our comprehensive analysis using hyphenated metabolomics platforms, MPK4 mutation affected arginine metabolic pathways. In addition to being an essential amino acid for protein synthesis, arginine is also a precursor for specialized metabolites, such as polyamines. Polyamines, including putrescine, spermidine, and spermine, are involved in plant development [36,37], biotic stress [38,39], and abiotic stress response [40]. Among them, spermine is identified as a positive regulator of plant immunity. It enhances plant resistance to *Pseudomonas viridiflava*, *P. syringae*, and *Hyaloperonospora arabidopidis* [41,42]. Putrescine, spermidine, and spermine all had significant changes in the *mpk4* mutant. Spermine showed a decrease, while spermidine and putrescine increased in the *mpk4* compared to WT (Figure 4F). This observation coincides with the *Arabidopsis* wounding response where putrescine increases and spermine decreases [39]. In addition, in both cases, the mRNA levels of an arginine carboxylase (*ADC2*, *At4g34710*), catalyzing arginine into agmatine, increased [39,43]. This could be an explanation of the decreased arginine content and increased downstream putrescine.

The changes of JA in the *mpk4* were also similar to those in the wounding response [44]. JA and MeJA, as well as their upstream metabolites involved in alpha-linolenic acid metabolism, all increased in the *mpk4* mutant (Figure 4D). Three enzymes found in our proteomics data involved in JA biosynthesis showed the same trends of changes, i.e., ALLENE OXIDE SYNTHASE (AOS, *AT5G42650*) and LIPOXYGENASE 2 (LOX2, *AT3G45140*) increased, but PEROXISOME DEFECTIVE 1 (PED1, *AT2G33150*) decreased [45].

Salicylic acid also increased in *mpk4*, which is consistent with the previous result [18]. An upstream metabolite shikimate and two enzymes, ISOCHORISMATE SYNTHASE 1 (ICS1, *AT1G74710*) and a 4-substituted benzoates-glutamate ligase (PBS3, *AT5G13320*), also increased in the *mpk4* [43]. These two enzymes catalyze reactions from chorismite to isochorismoyl-glutamate, while chorismite is the end-product of the shikimate pathway and isochorismoyl-glutamate is the direct precursor of salicylic acid. SA suppresses JA through SA receptor NPR1 [46]. However, both SA and JA can accumulate at the early stage of ETI, and SA is required in the early stage of the JA signaling pathway through salicylic receptor NPR3 (*AT5G45110*) and NPR4 (*AT4G19660*), before sufficient SA begins to suppress JA responsive genes [47]. In our result, although both SA and JA were increased, SA increased by 7.4-fold, while JA increased by 2.4-fold. In summary, SA was increased in the *mpk4* mutant and began to trigger JA accumulation. The fold change of SA was larger than JA, so SA was still dominant in the *mpk4* mutant.

Spermine is independent of SA meaning that, although both spermine and SA accumulate during pathogen infection and induce pathogenesis-related (PR) protein accumulation, exogenous spermine cannot induce the accumulation of SA and vice versa [38]. However, spermine positively affects the JA signaling pathway as the increase of spermine promotes the expression of JA biosynthesis and JA-dependent defense genes, such as *AOS*, *LOX*2, and *JASMONATE-ZIM-DOMAIN PROTEIN 1* (*JAZ1*, *At1g19180*) [41,42]. In the *mpk4* mutant, spermine decreased, while JA and JA-related metabolites increased [48]. In summary, spermine appears to be a double-edged sword, connecting to MPK4 in fine tuning the levels of JA and related metabolites.

### 3.3. MPK4 Is Related with Chloroplast Integrity and Photosynthesis

In addition to arginine metabolism, JA and SA changes, chlorophyll content, and light-harvesting chlorophyll protein complex (LHC) components (including LHCA4 (*AT3G47470*), Lhb1B1 *(AT2G34430*), Lhb1B2 (*AT2G34420*), and LHCB6 (*AT1G15820*)) were all decreased in the *mpk4* mutant. However, photosystem subunits (PS), including PSID1 (*AT4G02770*), PSIN (*AT5G64040*), and PSBO1 (*AT5G66570*), were increased (Figure 4D). There were more decreased proteins from carbon fixation in the *mpk4* than increased ones, which is consistent with previous studies that chloroplasts of the *mpk4* had less starch granules [9]. In addition, an abnormal chloroplast structure was observed with an increasing number of plastoglobuli in the *mpk4* [9]. Plastoglobuli lipoprotein particles presenting in chloroplast plastids facilitate metabolite exchange with thylakoid, and play roles in chloroplast biogenesis, stress responses, and thylakoid breakdown. Plastoglobuli size increased during stress and developmental transitions [49]. There were 11 proteins located in plastoglobuli that increased in the *mpk4*, including AOS, fructose-bisphosphate aldolase (FBA1, *AT2G21330*; FBA2, *AT4G38970*), rubisco activase (RCA, *AT2G39730*), and CLP protease proteolytic subunit 2 (CLPR2, *AT1G12410*). Among them, CLPR2 is required for chloroplast and plastoglobuli formation [50], which may explain the increase of plastoglobuli and PSs, while three plastid-lipid-associated proteins (PAP), PAP2 (*AT4G22240*), PAP3 (*AT2G35490*), and PAP6 (*AT3G23400*), decreased. Reduced PAP2 and PAP3 can lead to reduced growth and increased oxidative stress [51]. Reduced PAP6 may increase sensitivity to ozone and ROS [52]. These changes may contribute to phenotype of the *mpk4* mutant, which was shown to be sensitive to ozone and ROS [17,34].

In this study, we have discovered metabolites, proteins, and pathways perturbed by the mutation of *MPK4*, but how MPK4 affects the aforementioned metabolic pathways is not known. Interestingly, the proteins mentioned above, including ADC2, AOS, LOX2, LHCA4, Lhb1B1, Lhbf1B2, PSID1, and CLPR2, all have the typical SP phosphorylation motif, so they could be potential targets for MPK4 phosphorylation [53,54]. Thus, functional characterization of the bona fide MPK4 substrates in the metabolic networks is an important research direction.

## 4. Materials and Methods

### 4.1. Plant Materials

Both WT *A. thaliana* Col-0 and heterozygous *mpk4* mutant (*SALK_056245*) seeds (obtained from Arabidopsis Biological Resource Center, Ohio, OH, USA) were soaked in 70% ethanol with 0.1% Tween 20 for 1 min. Then excessive water was removed with 90% ethanol. Seeds were dried on filter paper for 1 h. Dried seeds were then placed on a half-strength Murashige and Skoog medium (1/2 MS salt, 1% sucrose, 1% agar, pH 5.7). After vernalization at 4 °C for 2 days in the dark, seeds were germinated and grown in a growth chamber at 22/20 °C with a 8/16 h short-day light cycle (day/night). The light was white light and intensity was 160 µmol/(m^2^s). Three-week-old seedlings were transferred to soil in the same growth chamber. Homozygous *mpk4* mutant plants were selected based on its dwarf and curling leaf phenotype as well as transcript analysis using semi-qPCR. Leaves from five-week-old plants were used for metabolomics and proteomics.

### 4.2. Semi-qPCR Analysis of MPK4 Transcript

Total RNA was extracted from five-week-old plant leaves with an RNeasy Plant Mini Kit (QIAGEN, Germantown, MD, USA). A PrimeScript RT reagent Kit (New England Biolabs, Ipswich, MA, USA) was used for cDNA synthesis. A DreamTaq PCR Master Mix (2X) (Thermo Scientific, CA, USA) was used for qRT-PCR with a CFX96 171 Touch™ Real-Time PCR Detection System (Bio-Rad, Hercules, CA, USA). ACTIN2 was used as the internal reference. The primers were: MPK4 F: TGCTCTGAATACACAGCAGC, MPK4 R: CACACTGAGTCTTGAGGATTG; ACTIN2 F: CGTACAACCGGTATTGTGCTG, ACTIN2 R: AGTAAGGTCACGTCCAGCAAG. The PCR products were run through 1% agarose gel with 0.2 μg/mL ethidium bromide under 110 V for 30 min. A 100 bp DNA Ladder (NEB N3231S) was used as a size indicator.

### 4.3. Metabolite Extraction from WT and Mpk4 Leaves

Metabolite extraction was conducted according to a previous publication [55] with minor modifications. Briefly, 300 mg fresh weight leaves from five-week-old plants were put in Eppendorf tubes and immediately frozen in liquid nitrogen. Samples were ground into fine powder with a plastic pestle in the presence of liquid nitrogen. Then, 0.1 mM lidocaine and camphorsulfonic acid were spiked into samples as an internal standard for a positive mode and negative mode, respectively. Metabolites were sequentially extracted with solvent I (Acetonitrile/Isopropanol/H_2_O = 3:3:2 by volume) and solvent II (Acetonitrile/H_2_O = 1:1 by volume). The resulting supernatants were combined and then lyophilized. The metabolites were resuspended in 50 µL 0.1% formic acid for LC-MS analysis. Four biological replicates were collected for each genotype.

### 4.4. Protein Extraction from WT and Mpk4 Leaves and Trypsin Digestion

The pellets left from the above metabolite extraction were used for protein extraction with a phenol method [56]. Briefly, proteins were extracted with the same volumes of an extraction buffer (0.9 M sucrose, 0.1 M Tris-HCl pH = 8.8, 10 mM EDTA disodium salt, 0.4% β-Mercaptoethanol and 2 mM phenylmethylsulfonyl fluoride) and Tris saturated phenol (pH = 8.8). After centrifugation at 10,000× *g* for 30 min, the phenol layers were mixed with 5 volume 0.1 M ammonium acetate in 100% methanol to precipitate the proteins. Protein pellets were washed once with 80% acetone and once with 100% acetone. The protein pellets were dissolved in 100 mM ammonium bicarbonate for in-solution trypsin digestion. The samples were treated with 10 mM dithiothreitol for 1 h at room temperature and 55 mM chloroacetamide in the dark for 1 h at room temperature. Then, proteins were digested with 200 ng/ul trypsin (Promega, Madison, WI, USA) in 50 mM ABC overnight at 37 °C. Tryptic peptides were desalted with ZipTip using the manufacture protocol (MilliporeSigma, Temecula, CA, USA). The peptides were lyophilized to dryness at 160 mBar using a Labconco CentriVap (Labconco Inc., Kansas City, MO, USA). Peptides derived from protein digests were resuspended in 0.1% formic acid (FA) for LC-MS analysis. Four biological replicates for each genotype were prepared for proteomics.

### 4.5. Liquid Chromatography Tandem Mass Spectrometry for Metabolomics and Proteomics

For targeted metabolomics, metabolite separation and mass spectrometry data acquisition were performed as previously described [57]. High performance liquid chromatography (HPLC)-multiple reaction monitoring (MRM)-MS/MS was conducted using an Agilent 1100 HPLC (Agilent, Santa Clara, CA, USA) coupled with an AB Sciex 4000 QTRAP^TM^ (AB Sciex, Framingham, MA, USA). Optimized detection conditions including precursor ion, product ion, declustering potential, collision energy, and cell exit potential were established for 338 authentic compounds including phytohormones. A reverse-phase C18 column (Agilent, Eclipse XDB-C18, 4.6 × 250 mm, 5 μm) was used for metabolite separation with 0.1% formic acid in water as solvent A and 0.1% formic acid in acetonitrile as solvent B. The LC gradient was initially held at 1% solvent B for 5 min, then a linear gradient was imposed from 1% B to 99.5% B over 41.5 min, followed by holding at 99.5% B for 4.5 min, and then return to 1% B. The flow rate was 0.5 mL/min. The MS conditions were: 30 psi for curtain gas, 50 psi GS1, 55 psi GS2, ion source voltage at 5500 V (for positive mode) and 4500 V (for negative mode), with the TurboIon electrospray interface temperature at 350 °C. The three periods in negative mode were separated at 15.9, 26.32, and 60 min. The five periods in the first positive mode method were separated at 5.8, 15.7, 23.7, 36.2, and 60 min. The three periods in the second positive mode method were separated at 20.0, 40.0, and 60 min.

For untargeted metabolomics with timsTOF^TM^, Bruker Elute ultra-high-performance LC (UHPLC) was connected to a Bruker timsTOF^TM^ instrument equipped with trapped ion mobility coupled to a hybrid quadrupole, time-of-flight mass spectrometer. Data acquisition was performed as previously described [29]. Samples were loaded onto an Accucore C18 column (100 mm × 2.1 mm, 2 μm). The column chamber temperature was 35 °C, and the pump flow rate was 0.4 mL/min. Solvent A (0.1% FA in water) and solvent B (0.1% FA in 95% acetonitrile) were used. Separation was conducted using the following gradient: 0–40% of B over 0–21 min; 40–95% of B over 21–23 min and isocratic at 95% of B over 23–24 min; then from 95–0% of B from 24–25 min and stop at 30 min. Ions were generated in both positive and negative electrospray ionization mode. The ESI used a 4500 V capillary voltage, and a 3.0 bar nebulizer pressure. The TIMS cartridge tunnel in pressure was set to 2.50 mbar and tunnel out pressure was 0.74 mbar. The TIMS ion funnels used 275–350 Vpp amplitude depending on the analytes mass. Our metabolomics analyses used a funnel 1 RF of 250 Vpp. TIMS inverse reduced mobility (1/K0) data were collected over a range of 0.4–1.8 1/K0. To prevent TIMS tunnel saturation and space charge effects during TIMS analyses, the ion charge control (ICC) software option was used to adjust the accumulation time of the ions in the first segment of the TIMS, and the ICC set between 2–5 million (Mio). Mass spectral data were collected on the QTOF-MS using a scan range of m/z 100–900. Untargeted metabolomics data were also acquired on Vanquish™ Horizon UHPLC Systems (Thermo Fisher Scientific Inc., San Jose, CA, USA) coupled with a Q Exactive^TM^ Plus Orbitrap Mass Spectrometer (Thermo Fisher Scientific Inc., Bremen, Germany). The LC conditions were the same as those for the timsTOF above. For MS data acquisition, full MS1 scan (m/z 60–900) was performed in the Orbitrap mass analyzer with a resolution of 70,000. The automatic gain control (AGC) target of 1e6 with 100 ms as maximum injection time (MIT). The dd-MS2 scan used 1 microscan, a resolution of 17,500, an AGC target of 5e5, an MIT of 46 ms, and a loop count of 3.

Proteomics data was acquired on an UltiMate™ 3000 RSLCnano system (Thermo Fisher Scientific Inc., Germering, Germany) coupled with a Q Exactive^TM^ Plus Orbitrap Mass Spectrometer (Thermo Fisher Scientific Inc., Bremen, Germany). Samples were loaded onto an Acclaim PepMap^®^ 100 C18 trapping column (75 μm i.d. × 2 cm, 3 μm, 100 Å) and then separated on a PepMap^®^ C18 analytical column (75 μm i.d. × 25 cm, 2 μm, 100 Å). The flow rate was set at 300 nL/min with solvent A (0.1% FA in water) and solvent B (0.1% FA in ACN) as the mobile phase. Separation was conducted using the following gradient: 0–2% of B over 0–8 min; 2–35% of B over 8–140 min; 35–95% of B over 140–160 min, and isocratic at 95% of B over 160–165 min, and then from 95–2% of B from 165–170 min. The equilibration at 2% is from 170 to 180 min. For MS data acquisition, the full MS1 scan (*m*/*z* 400–2000) was performed in the Orbitrap with a resolution of 70,000. The automatic gain control (AGC) target is 1e6 with 100 ms as the MIT. Peptides bearing 2 to 5 charges were selected with an intensity threshold of 9.1e5. Dynamic exclusion of 60 s was used to prevent resampling the high abundance peptides. The MS/MS was carried out in the Orbitrap with a quadrupole isolation window of 1.3. Fragmentation of the top 10 selected peptides by high energy collision dissociation (HCD) was done at 30% of normalized collision energy. The MS2 spectra were acquired at a resolution of 17,500 with an AGC target of 5e5 and an MIT of 55 ms.

### 4.6. Data Analysis

For targeted metabolomics, MultiQuant^TM^ software (version 2.1, AB Sciex, Foster City, CA, USA) was used to integrate chromatographic peak areas and quantitative analysis. The smoothing points were set at 2, noise percentage at 40%, peak splitting at 3 points, and baseline subwindow at 2 min. The processed data were manually reviewed for quality of integration and compared against authentic standard peaks. For untargeted metabolomics, raw data from timsTOF™ were processed through MetaboScape2021^®^ (Bruker, Bremen, Germany) against Bruker Sumner MetaboBASE Plant library and Bruker MetaboBASE Personal Library. In result filter section, min. number of features for extraction was set to 2 and min. number of featutes for result was set to 2. Eic correlation for ion deconvolution was set to 0.8 Raw data from the Orbitrap were analyzed using Coumpound Discoverer^TM^ (version 3.1, Thermo Thermo Scientific, Bremen, Germany) against mzCloud^TM^, ChemSpider and Metabolika database. Data were normalized against the internal standards. Metabolomic data from three platforms were combined. Redundant metabolites were compared between platforms based on their CV. Those with smaller CV were used for quantitative analysis.

Proteome Discoverer^TM^ (version 2.5, Thermo Scientific, Bremen, Germany) was used to search the MS/MS spectra from the protein samples. The SEQUEST algorithm in the Proteome Discoverer was used to process raw data files. Spectra were searched using the TAIR10 protein database with the following parameters: 10 ppm mass tolerance for MS1 and 0.02 as mass tolerance for MS2, two maximum missed tryptic cleavage sites, a fixed modification of carbamidomethylation (+57.021) on cysteine residues, dynamic modifications of oxidation of methionine (+15.996). Search results were filtered at 1% false discovery rate (FDR) and at least two unique peptides per protein for protein identification. Relative protein abundance in the samples was measured using label-free quantification. Proteins identified and quantified in all the four biological samples were used, and no imputation was performed. Peptides in samples were quantified as the area under the chromatogram peak. FDR cutoffs for both peptide and protein identification were set as 1%. Data were subjected to log10 transformation for normal distribution. Coefficient of variance (CV) was calculated and those with CV smaller than 20% were used for quantitative analysis.

Normalized datasets were further subjected to a *t*-test, principal component analysis, and volcano plot using MetaboAnalyst R package (https://www.metaboanalyst.ca (accessed on 23 June 2021)) [58]. GO analysis and KEGG pathway analysis were conducted using DAVID (https://david.ncifcrf.gov (accessed on 5 January 2022)) [59].

## 5. Conclusions

In this study, we integrate both metabolomics and proteomics data of WT and the *mpk4*. By analyzing the differential proteins and metabolites, we found that MPK4 was related with multiple pathways, including arginine biosynthesis and metabolism, photosynthesis and salicylic biosynthesis. The findings improve understanding between MPK4 molecular function and phenotypical traits and provide a potential network model for MPK4 function.

## Data Availability

The datasets presented in this study can be found in online repositories. The names of the repository/repositories and accession number(s) can be found below: The proteomics raw data and search results have been deposited to the ProteomeXchange Consortium via the PRIDE partner repository with the data set identifier PXD030175 (Reviewer account details: Username: reviewer_pxd030175@ebi.ac.uk; Password: 648LtXWk). The metabolomics raw data have been deposited to the Metabolights data repsitory with the data set identifier MTBLS3922.

## Supplementary Materials

The following are available online at https://www.mdpi.com/article/10.3390/ijms23020880/s1.
