# Supplementary figures and images for: Mitogen-Activated Protein Kinase 4-Regulated Metabolic Networks
Source: Int J Mol Sci. 2022 Jan 14;23(2):880. doi: 10.3390/ijms23020880 (PMC8779387; doi:10.3390/ijms23020880)

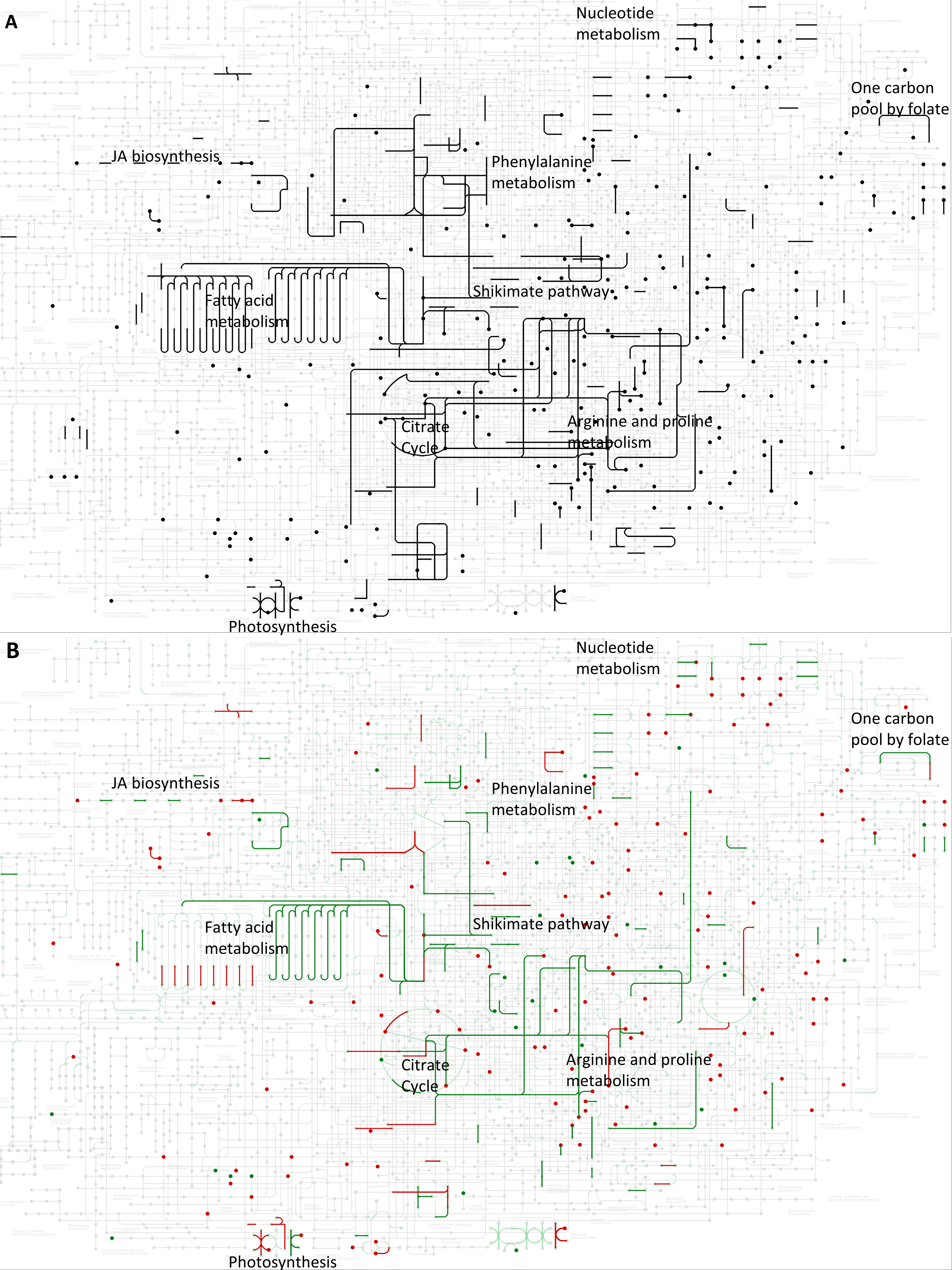

Supplement: Supplementary file 1 [file ijms-23-00880-s001.zip › Supplemental Figure S1.tif]
